# Assessing the exposure of forest habitat types to projected climate change—Implications for Bavarian protected areas

**DOI:** 10.1002/ece3.5877

**Published:** 2019-11-28

**Authors:** Claudia Steinacker, Carl Beierkuhnlein, Anja Jaeschke

**Affiliations:** ^1^ Department of Biogeography University of Bayreuth Bayreuth Germany; ^2^ Bayreuth Center for Ecology and Environmental Research BayCEER Bayreuth Germany; ^3^ Geographical Institute Bayreuth GIB Bayreuth Germany

**Keywords:** climate change impacts, conservation, ecosystems, European Union, forests, Habitats Directive, Natura 2000, sensitivity, species distribution models, vulnerability

## Abstract

**Aim:**

Due to their longevity and structure, forest ecosystems are particularly affected by climate change with consequences for their biodiversity, functioning, and services to mankind. In the European Union (EU), natural and seminatural forests are protected by the Habitats Directive and the Natura 2000 network. This study aimed to assess the exposure of three legally defined forest habitat types to climate change, namely (a) *Tilio‐Acerion* forests of slopes, screes, and ravines (9180*), (b) bog woodlands (91D0*), and (c) alluvial forests with *Alnus glutinosa* and *Fraxinus excelsior* (91E0*). We analyzed possible changes in their Bavarian distribution, including their potential future coverage by Natura 2000 sites. We hypothesized that protected areas (PAs) with larger elevational ranges will remain suitable for the forests as they allow for altitudinal distribution shifts.

**Methods:**

To estimate changes in range size and coverage by PAs, we combined correlative species distribution models (SDMs) with spatial analyses. Ensembles of SDM‐algorithms were applied to two climate change scenarios (RCP4.5 and RCP8.5) of the HadGEM2‐ES model for the period 2061–2080.

**Results:**

Our results revealed that bog woodlands experience the highest range losses (>2/3) and lowest PA coverage (max. 15% of sites with suitable conditions). *Tilio‐Acerion* forests exhibit opposing trends depending on the scenario, while alluvial forests are less exposed to climatic changes. As expected, the impacts of climate change are more pronounced under the “business as usual” scenario (RCP8.5). Additionally, PAs in flat landscapes are more likely to lose environmental suitability for currently established forest habitat types.

**Main conclusions:**

Based on these findings, we advocate the expansion of the Natura 2000 network particularly in consideration of elevational gradients, connectivity, and projected climatic suitability. Nonclimatic stressors on forest ecosystems, especially bog woodlands, should be decreased and climate change mitigation efforts enhanced. We recommend transferring the approach to other habitat types and regions.

## Graphical abstract

Climate change particularly affects forest ecosystems due to their longevity and slow migration response. Using ensembles of species distribution models, this study analyzed the exposure of forest habitat types from the EU Habitats Directive to projected climate change. Focussing on their conservation status, the environmental suitability of Natura 2000 sites was modeled in order to detect future short‐comings of the protected area network on a regional scale. The results show profound differences in exposure among the assessed forest habitat types, as well as between climate change scenarios.

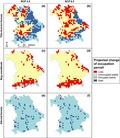

## 1 INTRODUCTION

Besides timber and fuel production, forests are providing a series of ecosystem services for human well‐being and societal interests (Brockerhoff et al., 2017). Forest ecosystems play a crucial rule, inter alia, for carbon sequestration, balancing climatic extremes and maintaining biodiversity, explaining their importance in nature conservation and for protected area networks.

In the European Union (EU), natural and seminatural forest habitat types are listed in the Habitats Directive since 1992 (European Council, 1992). In this Directive, standardized “habitat types” are defined by characteristic species assemblages and abiotic conditions. One instrument, which arose from this EU legislation, is the Natura 2000 network. It was set up as a Europe‐wide network of conservation sites, which were designated under the Habitats Directive and the Birds Directive from 1979 (European Council, 1992). Covering more than one‐fifth of the EU territory, Natura 2000 is declared the largest protected area network across the globe (EEA, 2015).

However, concerns have been issued whether static protected area (PA) networks will represent the species and habitats of conservation interest under projected climate change (e.g., Alagador, Cerdeira, & Araújo, 2016; Hannah, 2008; Kujala, Araújo, Thuiller, & Cabeza, 2011). According to a study by Araújo, Alagador, Cabeza, Nogués‐Bravo, and Thuiller (2011), more than half of the assessed species from the EU Habitats and Birds Directives will lose suitable climatic conditions within current European PAs until 2080. One‐fifth of the listed habitats is graded as threatened by climate change according to the countries’ reports (Evans, 2012).

Forests are considered to exhibit particularly fragile habitat types (Evans, 2012; Wagner‐Lücker, Förster, & Janauer, 2014). Both, their measured conservation status and projected distribution losses are worse in comparison with other habitats (Dempe, Jaeschke, Bittner, & Beierkuhnlein, 2012; EEA, 2015). Their long life spans, slow migration responses, and the fragmentation of landscapes impede necessary distribution shifts to follow suitable climate (e.g., Honnay et al., 2002; Lindner et al., 2014; Milad, Schaich, Bürgi, & Konold, 2011; Renwick & Rocca, 2015; Zhu, Woodall, & Clark, 2012). We argue that the spatial responses of forest habitat types to climatic changes have not received enough attention in research despite their high climate‐sensitivity, large share within Natura 2000 areas, and important role as carbon sinks (EEA, 2016; Orlikowska, Roberge, Blicharska, & Mikusiński, 2016). Studies on climate change impacts on European forests have either focussed on individual tree species (Buras & Menzel, 2019; Dyderski, Paź, Frelich, & Jagodziński, 2018; Frejaville, Fady, Kremer, Ducousso, & Garzon, 2019) or, less frequently, on regional forest ecosystems (Hester, Britton, Hewison, Ross, & Potts, 2019; Lehsten et al., 2015). Plant communities have rarely been addressed and do not relate directly to the habitat types of the Habitats Directive.

This study addresses three EU forest habitat types (official EU code in brackets) spanning across a wide range of site conditions:

- *Tilio‐Acerion* forests of slopes, screes, and ravines (9180*)
- Bog woodlands (91D0*)
- Alluvial forests with *Alnus glutinosa* and *Fraxinus excelsior* (91E0*).

Within Annex I of the EU Habitats Directive, they are categorized as priority (indicated by asterisk in their codes). Priority natural habitat types are classified as “in danger of disappearance,” which translates into enhanced conservation responsibilities for the member states (European Council, 1992). Therefore, it is of outstanding importance to investigate menaces to their persistence. Climate change represents a major threat to the selected forest habitat types as warming combined with drought is expected to cause water stress in plants (Breshears et al., 2013). All types of forest in this study are soil moisture dependent (EC, 2013)—either by their vicinity to rivers and shallow aquifers (alluvial forests), their substrate and precipitation regime (bog woodlands) or their shady topography and reduced evapotranspiration (*Tilio‐Acerion* forests). The restriction of these forests to specific site conditions renders them especially vulnerable to climate change, as they might not find the required conditions in places with future climatic suitability.

The study was carried out for the federal state of Bavaria in Germany. The responsibilities for nature conservation legislation and implementation, including of EU Directives, lie at this administrative level. Consequently, Natura 2000 sites are designated by regional authorities and the habitat types monitored at this scale. The example of Bavaria was chosen because of its extensive area of woodland (about 2.6 million hectares, approx. 37% of the total area of Bavaria) ranging across a diversity of landscapes and elevation (BMEL, 2015). Bavaria has designated 11.4% of its territory to Natura 2000 sites (LfU, 2018). The individual Natura 2000 sites vary significantly in their spatial extent—from very local features to national parks, former military areas, and vast mountain ranges. Bavaria is located between continental and alpine biogeographic regions of Europe and represents a transition zone of contrasting future rainfall trends (Jacob et al., 2014; Kovats et al., 2014; Stagl, Hattermann, & Vohland, 2015). Although the predicted precipitation patterns vary geographically within the region and between climate models (Stagl et al., 2015; Wagner, Berg, Schädler, & Kunstmann, 2013), studies mostly agree on decreases in summer season and gains in winter (Gerstengarbe, Hoffmann, Österle, & Werner, 2015; Pfeifer et al., 2015). These forecasts have severe implications for forests, as they would limit the water available to plants during the vegetation period. Regarding the future temperature development, regional climate models predict increases of varying magnitude (e.g., Jacob et al., 2014). In the Alpine foothills, for example, summer warming is expected to exceed 4°C in comparison with 1971–2000 by the end of this century under an extreme scenario (Jacob et al., 2014).

To analyze climate change threats to the selected forest habitat types, this study built on ensembles of correlative species distribution models (SDMs), which are widely used in climate change impact research and conservation planning (e.g., Meller et al., 2014; Summers, Bryan, Crossman, & Meyer, 2012). For conservation purposes, it is crucial to communicate the multitude of possible trajectories to avoid decision‐making based on a singular, uncertain model projection. Ensembles of model forecasts are capable of illustrating the range and trend of projections, which vary across algorithms, model setting, global circulation models, or climate change scenarios (Araújo & New, 2007).

Using this methodology, we aimed to: (a) predict changes in the habitat types’ range size and distribution, (b) estimate their potential future coverage by PAs, (c) and examine the linkage between elevational ranges within PAs and their projected environmental suitability for the considered forest habitat types. Previous studies have documented distribution shifts of tree species along elevational gradients (both upward and disparate directions) under warming climate (e.g., Morin et al., 2018; Rabasa et al., 2013). Therefore, we hypothesized that PAs with larger elevational ranges are more likely to remain hosts of currently established forests as they allow for such distribution shifts. The analyses of this study were designed to enable comparisons between habitat types with regard to the impacts of climate change in order to detect eventual needs for management and adaptation strategies.

## 2 METHODS

### 2.1 Distribution data and environmental predictors

The three habitat types, which are defined in the EU Habitats Directive, were selected in order to represent a range of forest ecosystems. (a) *Tilio‐Acerion* forests of slopes, screes, and ravines occur on locations with steep topography as indicated in their name. They are divided into two groups: the dry and warm environments with lime trees (*Tilia cordata*, *Tilia platyphyllos*) and the humid and cool sites with sycamore maple (*Acer pseudoplatanus*) dominance. (b) Bog woodlands depend on oligotrophic, wet, or humid peat substrates with high groundwater table. Both coniferous and broad‐leaved tree species can be found under this habitat type. (c) Alluvial forests with *Alnus glutinosa* and *Fraxinus excelsior* develop along rivers or on other periodically inundated soils. Three phytosociological vegetation units can be distinguished: *Alno*‐*Padion*, *Alnion incanae,* and *Salicion albae*. Detailed descriptions of the habitat types can be found in the official EU manual (EC, 2013).

**Figure 1:**
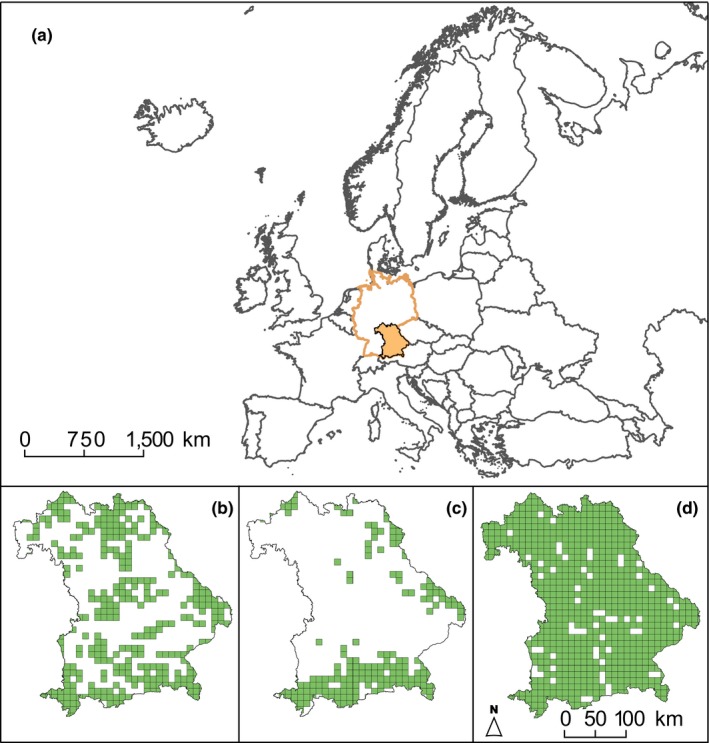
Current distribution of important moisture‐dependent forest habitat types in Bavaria, Germany. Terminology is given by the EU Habitats Directive. (a) Location of study region within Europe (orange filling = Bavaria, orange outline = Germany). (b) “*Tilio‐Acerion* forests of slopes, screes, and ravines,” (c) “Bog woodlands,” and (d) “Alluvial forests with *A. glutinosa* and *F. excelsior.”* Occurrence data originate from mandatory monitoring of protected habitat types by member states between 2007 and 2012 (EEA, 2018). Records are protocolled in 10 × 10 km grid cells

We applied a direct modeling approach to the habitat types, in which they are modeled in their entirety and not by means of their constituting species (sensu Bittner, Jaeschke, Reineking, & Beierkuhnlein, 2011). Necessary information on the distribution of the forest habitat types (Figure 1 and Figure S1.1) builds on the official reporting by EU member states for the period 2007–2012. In the monitoring reports, each 10 × 10 km grid cell, which harbors a forest patch, is marked as occurrence. For further analyses, centroids were extracted from the reported occurrences. Occurrence data were considered for the continental scale to cover wider ecological niches in the models than the mere records within Bavaria would depict (following Falk & Mellert, 2011).

Climatic variables, soil attributes, and topographic information were included as environmental predictors for the correlative species distribution modeling. The ecological needs of the key tree species (EC, 2013; IUCN, 2018) were considered for the identification of relevant variables for each habitat type (Table 1). Additionally, correlation between predictors was excluded (Pearson correlation coefficient >0.7, sensu Bittner et al., 2011) and variable importance measured with the R package “hier.part” (Walsh & Mac Nally, 2015). The detailed procedure is shown in Appendix S2. The selected climate variables depict seasonal patterns and drivers of plant growth and health. To assess uncertainties related to climate development in the upcoming decades, two Representative Concentration Pathway (RCP) scenarios were used for the period 2061–2080: the moderate RCP4.5 and the “business as usual” scenario RCP8.5. The future climate data are based on the HadGEM2‐ES model, a successor of the widely used HadCM3 (Martin et al., 2011).

**Table 1:** Selected environmental predictors for forest habitat types utilized in correlative species distribution models

Environmental variables	Habitat types		
	*Tilio‐Acerion* forests	Bog woodlands	Alluvial forests
Minimum temperature of the coldest month	x	x	
Temperature annual range	x	x	x
Mean temperature of the wettest quarter	x	x	x
Mean temperature of the coldest quarter			x
Precipitation seasonality	x		x
Precipitation of the driest quarter	x		
Precipitation of the warmest quarter		x	x
pH in 2 m soil depth	x	x	x
Organic carbon content (g/kg) in 2 m soil depth		x	x
Elevation	x	x	x
Slope	x	x	

Note

'x' indicates, which variables were used for the ensemble model of each habitat type.

Climatic variables are derived from WorldClim (2016, n.d.), soil attributes from the ISRIC—World Soil Information institute (2018a, 2018b). The variable slope was calculated based on a digital elevation map provided by the European Environment Agency (EEA, 2017). All data sources and original resolutions are listed in Table S1.2.

### 2.2 Modeling

Ensembles of species distribution models (SDMs) were created with the “biomod2”‐package version 3.3‐7 (Thuiller, Damien, Engler, & Breiner, 2016) in R software 3.5.1 (R Core Team, 2018) to minimize inaccuracies of individual model algorithms. Generalized linear models (GLM), generalized additive models (GAM), generalized boosted methods (GBM), and random forest (RF) were integrated into the ensembles. Modeling took place at the 30‐arc‐seconds resolution of the utilized climatic variables. After masking out the 10 × 10 km occurrence cells, pseudo‐absences were selected randomly within the EU following the 50% prevalence approach by Liu, Berry, Dawson, and Pearson (2005). Input datasets were split into train (70%) and test data (30%) for the evaluation of the models’ performances. In a cross‐validation process, the validation measures ROC (relative operating characteristic), TSS (true skill statistic), and Cohen's Kappa were determined. The model outputs were projected to both current and future environmental conditions of Bavaria. Finally, a total consensus model was compiled over all model algorithms and runs for each habitat type. Following Marmion, Parviainen, Luoto, Heikkinen, and Thuiller (2009), we used the weighted mean of the ensembles for further analysis. Using the threshold that maximizes the sum of sensitivity and specificity (Liu, White, & Newell, 2013), the occurrence probabilities for each habitat type were converted into binary information.

### 2.3 Range change, coverage analysis, and elevational gradient statistics

Based on the modeling results, range changes of the habitat types were estimated for each RCP scenario by comparing the projected future distribution with reported present‐day occurrences (“BIOMOD_RangeSize”‐function in “biomod2”‐package). For this purpose, the binary model outputs (30 arc‐seconds) were aggregated to the resolution of the original distribution data (10 × 10 km). The estimated changes in range size for the study region served as one criterion for the determination of the habitat types’ exposure levels.

As second criterion for the exposure of habitat types to climate change, we assessed their coverage by PAs in Bavaria. The overlap of potential occurrences of the forests with shapefiles of Natura 2000 sites was computed for all time steps and RCP scenarios. The following categories express changing environmental suitability of the protected areas with respect to the forest habitat types:

- unoccupied stable: PA does not host habitat type at present nor in future;
- loss: PA hosts habitat type at present but potentially not in future;
- occupied stable: PA hosts habitat type at present and potentially also in future;
- gain: PA does not host habitat type at present but potentially in future.

The changing environmental suitability of the PAs was then tested against the elevational range found inside of the corresponding conservation site. The zonal statistics tool in ArcGIS 10.5 was used to assign elevation ranges to the individual PAs based on a digital elevation map (EEA, 2017). Kruskal–Wallis rank‐sum tests (R package “stats” 3.5.1) compared the previously defined “changing suitability” categories of the PAs with regard to this elevation range attribute. Post hoc tests were carried out with the “kruskalmc”‐function of the R package “pgirmess” version 1.6.9 (Giraudoux, Antonietti, Beale, Pleydell, & Treglia, 2018). Wilcoxon tests (R package “stats”) were executed to furthermore test whether the elevational range interferes with the future environmental suitability of PAs independent of their current occupation by the habitat types.

## 3 RESULTS

### 3.1 Range change of forest habitat types under climate change in Bavaria, Germany

**Figure 2:**
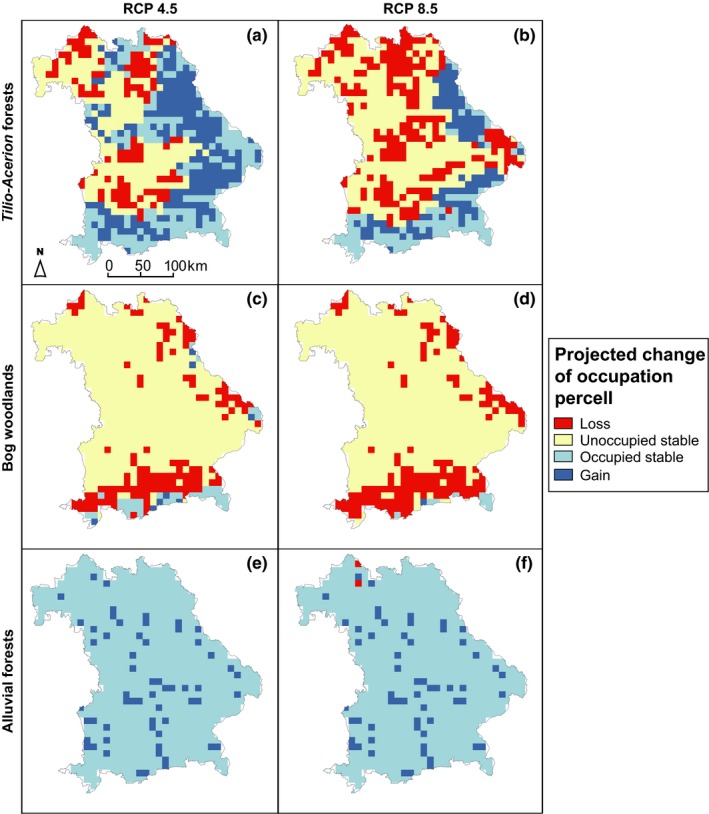
Modeled range change of (a, b) *Tilio‐Acerion* forests of slopes, screes, and ravines, (c, d) bog woodlands, and (e, f) alluvial forests with *A. glutinosa* and *F. excelsior* in Bavaria until 2061–2080. Change classes distinguish between cells, which are likely to lose the habitat type, gain suitability for it or remain either stable occupied or unoccupied by it under climate change. For detailed description on change analysis and models, see Section 2 and caption of Table 2

The three studied forest habitat types face very different perspectives until the 2070s. Bog woodlands react most sensitively to expected abiotic changes. Up to 94% of their small Bavarian range are projected to be lost until the second half of this century (Table 2). Irrespective of the scenario, <10% of the entire study region will provide suitable environmental conditions for bog woodlands. Potential refugia are situated in the Alps and their foothills (Figure 2). For RCP4.5, sites in eastern Bavaria additionally remain suitable or emerge as appropriate locations.

**Table 2:** Projected range change of *Tilio‐Acerion* forests of slopes, screes, and ravines (9180*), bog woodlands (91D0*), and alluvial forests with *A. glutinosa* and *F. excelsior* (91E0*) in Bavaria, Germany

		Climate change scenario	Total suitable grid cells [%]	Lost grid cells [%]	Gained grid cells [%]	Net change in occupied cells [%]
Habitat type	9180*	Current	41			
		RCP4.5	55	34	70	+36
		RCP8.5	28	67	35	−32
	91D0*	Current	19			
		RCP4.5	6	74	6	−68
		RCP8.5	1	94	<1	−94
	91E0*	Current	92			
		RCP4.5	100	0	9	+9
		RCP8.5	100	<1	9	+9

Note

Change analysis compared observed current with projected future occupation of cells (10 × 10 km). “Total suitable grid cells” refers to environmentally suitable proportion of Bavaria's territory. Percentages for “loss,” “gain,” “net change” are relative to current range size. The future distribution of the habitat types was modeled as ensembles of correlative species distribution models combining GAM, GLM, GBM and RF. Climate change scenarios RCP4.5 and RCP8.5 were considered for the HadGEM2‐ES model for 2061–2080.

For the *Tilio‐Acerion* forests, the spatial response varies considerably between the two climate change scenarios. Under RCP4.5, the model projects an overall range expansion (36% relative to current range size), constituted by both losses of currently occupied cells (34%) and gains of new suitable area (70%) (Table 2). In contrast, the proportion of newly available areas under RCP8.5 is noticeably lower (35% relative to current range size), while the projected losses amount to 67% of currently occupied grid cells. As a consequence, *Tilio‐Acerion* forests will experience an overall range contraction under RCP8.5 to about two‐third of their current range size within Bavaria. Spatially, the distribution of this habitat type will shift east‐ and southward (Figure 2) according to the model. While the observed presences in central and northern Bavaria could still be occupied under RCP4.5, they are likely to disappear under RCP8.5.

The alluvial forests with *A. glutinosa* and *F. excelsior* prove to be less exposed. For both climate change scenarios, this habitat type is projected to not shrink. Focussing on the fundamental environmental suitability of space rather than the exact locations of watercourses, the alluvial forests could slightly expand their range under RCP4.5 (9% gain relative to current range size) (Table 2).

### 3.2 Future coverage of forest habitat types in the Bavarian Natura 2000 network

One important criterion when assessing the endangerment of forest types is their coverage by PAs. The intersection between modeled future distribution of the forest habitat types and the locations of Bavarian Natura 2000 sites revealed distinct differences in potential representation (Table 3).

**Table 3:** Changing environmental suitability of Bavarian Natura 2000 sites to host habitat types *Tilio‐Acerion* forests of slopes, screes, and ravines (9180*), bog woodlands (91D0*), and alluvial forests with *A. glutinosa* and *F. excelsior* (91E0*) under climate change

		Climate change scenario	[%] of all Bavarian Natura 2000 sites				[%] of currently suitable Bavarian Natura 2000 sites	
			Unoccupied stable	Loss	Occupied stable	Gain	Loss	Occupied stable
Habitat type	9180*	RCP4.5	15	21	51	14	29	71
		RCP8.5	23	43	28	6	61	39
	91D0*	RCP4.5	58	27	14	<1	66	34
		RCP8.5	59	38	3	0	93	7
	91E0*	RCP4.5	0	0	98	2	0	100
		RCP8.5	0	<1	98	2	0	100

Note

Based on intersection of observed current and projected future distribution of habitat types with protected area (PA) polygons. For detailed description on models, see Section 2 and caption of Table 2. Change classes of PAs: "unoccupied stable" (no current host and no future host of habitat type), "loss" (current host of habitat type but no future host), "occupied stable" (current and future host of habitat type) or "gain" (no current host but future host of habitat type).

Even under the criterion of PA coverage, bog woodlands reach the highest exposure scores. Two‐thirds of the PAs, which accommodate the habitat type at present, lose their function as host even under moderate climate change (RCP4.5). For RCP8.5, this proportion raises to more than 90%. From all Bavarian Natura 2000 sites, 15% potentially accommodate fractions of bog woodlands under RCP4.5 and as little as 3% provide suitable environmental conditions under RCP8.5. Under RCP8.5, only 310 km^2^ of the modeled suitable territory fall within currently established PAs.

The projected future PA coverage of *Tilio‐Acerion* forests of slopes, screes, and ravines is likewise threatened. Twenty nine percent (RCP4.5) and 61% (RCP8.5) of those sites, which host the habitat type nowadays, lose their environmental suitability during the 21st century. The pronounced differences between climate change scenarios in the case of this habitat type are visible for the conservation status as well. Thirty four percent of all Bavarian Natura 2000 sites potentially intersect with *Tilio‐Acerion* forests under RCP8.5, compared to 65% under RCP4.5. Along with the projected range reduction under RCP8.5, the area suitable for *Tilio‐Acerion* forests and covered by PAs drops by 42%.

For alluvial forests with *A. glutinosa* and *F. excelsior*, more Natura 2000 sites than today seem to be capable of accommodating the habitat type in the future. For the change classes, neither “loss” nor “unoccupied stable” are represented in RCP4.5 and are negligible under RCP8.5.

### 3.3 Elevational range as predictor for future suitability of protected areas

**Figure 3:**
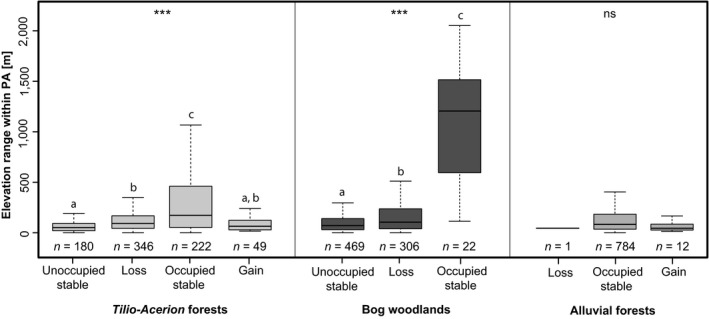
Local elevation range within Bavarian Natura 2000 areas in relation to their modeled function as potential hosts for selected forest habitat types under climate change scenario RCP8.5. For detailed description on change classes of PAs and models, see Section 2 and caption of Tables 2 and 3. Statistics were performed with Kruskal–Wallis rank‐sum test and “kruskalmc” post hoc test (R packages “stats” and “pgirmess”). Significance levels are expressed by asterisks, where *** symbolises p values of ≤ .001 and 'ns' refers to p values ≥ .05. The lowercase alphabets describe, which groups are significantly different from each other. Additional statistical graphs in Appendix S3

In addition to the exposure assessments for the forest habitat types, it was analyzed whether larger elevational ranges inside of conservation sites favor their future role as refuge for endangered habitat types. The statistical results (Figure 3, Appendix S3) indicate that PAs differ significantly in their future potential for hosting two out of three habitat types (bog woodlands and *Tilio‐Acerion* forests) depending on the elevational range found inside of them. Partially confirming the initial assumption, PAs with larger elevational ranges are more likely to maintain environmental suitability for these two habitat types for both climate change scenarios. For the widespread alluvial forests such a pattern could not be found.

### 3.4 Model evaluation

Evaluating the quality of the models, projections for the reference period of 1970–2000 were mostly over‐predictive in comparison with the currently observed distributions within Bavaria (compare Figure 1 and Appendix S4). On European scale, the ensemble models were capable to project the range of the forest habitat types adequately (Table S5.1 and Figure S1.1). Comparing expert‐based ecology descriptions for the key tree species (e.g., EC, 2013; IUCN, 2018) with generated response curves (Appendix S6) and variable importance rankings (Table S7.1) permitted an additional assessment of the models’ plausibility. Known differences of the forest types with regard to frost sensitivity, for example, were captured well by the models. The corresponding variables “minimum temperature of the coldest month” or “mean temperature of the coldest quarter” were crucial to explain current distribution patterns of all three habitat types. While bog woodlands tolerate cold temperatures, which is emphasized by their range expansion toward Northern Europe (Figure S1.1), *Tilio‐Acerion* forests and alluvial forests react sensitively toward frost. The different ecological requirements also serve to reason the reaction of the individual habitat types to climate change.

## 4 DISCUSSION

### 4.1 Exposure differences among forest habitat types

Measured by the modeled range size, range change and coverage by Natura 2000 areas, the investigated forest habitat types exhibit different levels of exposure toward climate change at a regional scale. Comparable previous research did not consider the criterion of protected area coverage. Nevertheless, literature supports the results presented here by likewise predicting range losses for many tree species and stressing sensitivity differences (Dyderski et al., 2018; Ohlemüller, Gritti, Sykes, & Thomas, 2006; Walentowski et al., 2017). In this context, altered species compositions of forests become relevant (Buras & Menzel, 2019). According to Schlumprecht, Gohlke, and Bierkuhnlein (2014), distribution shifts of habitat types are less likely than the contraction of their ranges. In our study, bog woodlands are most threatened, followed by *Tilio‐Acerion* forests of slopes, screes, and ravines. The alluvial forests with *A. glutinosa* and *F. excelsior* seem to be remarkably less exposed to climatic changes.

For bog woodlands, projects by the German Federal Agency for Nature Conservation (Bittner & Beierkuhnlein, 2014) and the European Topic Centre on Air and Climate Change (Otto, Harley, van Minnen, Pooley, & de Soye, 2012) also assigned high vulnerability levels to this habitat type. For *Picea abies* and *Pinus sylvestris*, which are characteristic to bog woodlands, other studies projected vast declines for their European range (Takolander, Hickler, Meller, & Cabeza, 2019) and their climate envelopes’ accordance with the climatic conditions of Bavaria (Kölling & Zimmermann, 2007). Based on the identified high importance of cold minimum temperatures for this habitat type, we conclude that expected milder temperatures cause the projected negative range trends of bog woodlands. Seasonal decreases in precipitation might be a further threat to bog woodlands, as they depend on specific soil water conditions (EC, 2013). Due to their low pH values and restrictions in nutrient availability (see Figure S6.2; Table S7.1), bog woodlands are additionally narrowed to sites that will not evolve within short time scales.

Focusing on the tree species of *Tilio‐Acerion* forests, a trade‐off between the thermophilic character of *T. cordata* and *T. platyphyllos* (Ellenberg & Leuschner, 2010) and the drought sensitivity of *T. cordata* and *A. pseudoplatanus* (Crowley, Rivers, & Barstow, 2017; Rivers, Barstow, & Khela, 2017) might explain the opposing trends in range size depending on the considered climate change scenario. While a moderate warming (especially of minimum temperatures) under RCP4.5 is likely to favor this habitat type, conditions under RCP8.5 might become too dry for its persistence (lower precipitation of the driest quarter). Discrepancies between our results and the studies by Kölling and Zimmermann (2007), as well as Bittner and Beierkuhnlein (2014), therefore potentially originate in differences of climate change scenarios, climate models, time period, and utilized variables. These studies found the region as a stable host for *Tilio‐Acerion* forests and identified a low susceptibility of key tree species to climate change. However, model results for single species cannot be translated directly into the development of the corresponding habitat type.

Investigating *A. glutinosa* and *F. excelsior*, belonging to the alluvial forests, Kölling and Zimmermann (2007) support the here postulated positive trend for the future. A potential reason for the projected high occurrence probability of alluvial forests within Bavaria might lie in milder mean temperatures of the coldest quarter. Both experts (Shaw, Roy, & Wilson, 2014) and our models highlight the sensitivity of alluvial forests to longer phases of frost. Note that in total three phytosociological alliances and a magnitude of plant species fall into the definition of this habitat type resulting in a high variety of species assemblages, which enlarges its tolerated spectrum of environmental conditions and aggravates interpretations in an ecological context. Including very different communities in its definition in EU legislation also explains the low sensitivity of this habitat type to climatic changes in comparison with others that are comparably uniform in species composition.

### 4.2 Implications for nature conservation

This study demonstrated that all examined forest habitat types face less favorable conditions under more intense climatic changes (i.e., RCP8.5). This relates to modeled changes in range size and future coverage by PAs. The estimated representation within the Bavarian Natura 2000 network is lower for all three habitat types under RCP8.5 than RCP4.5. For *Tilio‐Acerion* forests, the two climate change scenarios even cause opposite trends in range size development. Therefore, the mitigation of anthropogenic climate change must be the first step of any conservation strategy. As climatic changes are unavoidable to some extent (Kovats et al., 2014), scientists also call for the reduction of nonclimatic stressors on endangered forests (EEA, 2016).

Further implications for conservation practice relate to the extent and functionality of the Natura 2000 network. The designation of additional sites seems necessary to sustain the representation of the forest habitat types despite climate change induced distribution shifts. Spatially, conservation gaps might form in the east and south of the study region. Especially, the Alpine foothills and Alps serve as refugia to the two more threatened habitat types.

In the selection process for new Natura 2000 sites, connectivity, coherence, area, redundancy, and climate change concerns need to be considered (Hannah, 2008). Connecting corridors or stepping stones should link recent occurrences with regions of projected future suitability and allow for genetic exchange between populations to foster adaptation (e.g., Keeley et al., 2018; Nuñez et al., 2013). As elevational ranges demonstrably influence the future potential of PAs to host certain habitat types, we add the inclusion of elevational ranges to these recommendations. Other researchers have already mentioned diversity of topography and “topoclimate” as necessary criteria (Heller et al., 2015; Nadeau, Fuller, & Rosenblatt, 2015). PAs flexible in space and time represent another progressive conservation concept (Bull, Suttle, Singh, & Milner‐Gulland, 2013; Hannah, 2008). However, Milad et al. (2011) raise concerns about competing land use interests and established property structures in the context of PA designations. Numerous case studies have described conflicts during the implementation of Natura 2000 sites and their management (e.g., Beunen & de Vries, 2011; Campagnaro, Sitzia, Bridgewater, Evans, & Ellis, 2019; Crossey, Roßmeier, & Weber, 2019; Gallo, Malovrh, Laktić, De Meo, & Paletto, 2018; Kati et al., 2015; Paletto et al., 2019). They suggest to enable participatory approaches which involve local stakeholders, for example, foresters and land owners.

Particularly for tree species and forest habitats, assisted colonization is an option worth considering (e.g., Williams & Dumroese, 2013). Kreyling et al. (2011) summarize the advantages and risks connected to this conservation concept. In a forest context, this would include the support of better‐adapted tree species or provenances and increasing overall heterogeneity and diversity. With assisted migration, the barriers imposed by landscape fragmentation and limited dispersal abilities of tree species could be reduced. The improvement of the management of Natura 2000 sites plays another important role in safeguarding the priority habitat types (Geyer, Kreft, Jeltsch, & Ibisch, 2017). Conservation measures for the here considered forest habitat types include, for example, the restoration of natural hydrological conditions and the removal of exotic plants (Hughes, del Tánago, & Mountford, 2012; Stiftung Naturschutzfonds Brandenburg, n.d.).

In the context of changing environmental suitability for target habitat types, assessments of the functionality of individual Natura 2000 sites become relevant. While additional sites would ensure the future coverage of protected habitat types, prospective unsuitable PAs should be investigated with respect to their purpose. Here, we argue that the multifunctionality of Natura 2000 areas reduces their risk of losing the reason for existence. Questions arise whether the EU Habitats Directive provides the means to adapt conservation strategies and the protected area network as described above (Cliquet, 2014). As the future might foster entirely new, unexperienced species compositions (“novel ecosystems”), altered legal definitions of the habitat types could ensure the maintenance of the conservation status of natural and seminatural forests of Europe.

### 4.3 Limitations

While this research highlights the usefulness of correlative SDMs for conservation purposes, we recognize limitations to the presented approach. Foregoing general criticism of correlative SDMs (see Araújo et al., 2019; Araújo & Peterson, 2012; Ferrier & Guisan, 2006; Peterson, Cobos, & Jiménez‐García, 2018), modeling forest habitat types remains particularly challenging.

A major obstacle lies in the availability of data: Firstly, monitoring data for EU habitat types is only available at a 10 × 10 km resolution. However, Bavarian Natura 2000 sites are often smaller. Secondly, distribution data from non‐EU countries would be useful, as nature does not conform with political boundaries. In addition, habitat types are interpreted differently across countries and cannot be described by the mere sum of their constituting species (Berry, 2012; Bittner et al., 2011; Evans, 2012). Therefore, we applied a direct modeling approach to the legally defined habitats. For habitat types, which are defined more uniformly, another approach (e.g., Mücher, Hennekens, Bunce, Schaminée, & Schaepman, 2009) may be suitable as well. There, the distribution of a habitat is modeled in an indirect manner based on indicator plant species and ecological knowledge.

It should be borne in mind that the direct impact of climate change is not the only threat to EU habitat types. Altered forest health conditions and pest outbreaks (e.g., bark beetles, see Marini et al., 2017) are additional challenges. Of outstanding importance for the here investigated forest habitat types is the ash dieback caused by the fungus *Hymenoscyphus pseudoalbidus*, which infects *F. excelsior* (Pautasso, Aas, Queloz, & Holdenrieder, 2013). Consequently, the projected widespread environmental suitability for alluvial forests with *A. glutinosa* and *F. excelsior* cannot be translated into a worry‐free future. Habitat type 91E0* is furthermore severely threatened by the invasion of alien plant species (Campagnaro, Brundu, & Sitzia, 2018). Natura 2000 sites are, in general, vulnerable to biological invasions due to their less strict exclusion of human activities (Gallardo et al., 2017; Guerra, Baquero, Gutiérrez‐Arellano, & Nicola, 2018). These menaces need to be considered when interpreting the future conservation status of protected habitat types.

### 4.4 Outlook

Building on the here modeled future environmental suitability of Bavaria for the considered forest habitat types, precise locations for complementary PAs can be identified in a next step by including mask layers for land cover and watercourses. This step will be particularly crucial to increase the reliability of suitability maps for the alluvial forests. We advocate to consider additional climate models and scenarios in subsequent ensemble modeling studies to further reduce uncertainties related to the climate development itself (Buisson, Thuiller, Casajus, Lek, & Grenouillet, 2010; Peterson et al., 2018).

Moving ahead methodologically, the monitoring of habitats from the EU Directive needs to be conducted at a better resolution. To further improve the quality of input data, research on nonequilibrium states of the observed distributions of European forests should be strengthened (García‐Valdés, Zavala, Araújo, & Purves, 2013). In order to advance the models themselves, we call for the incorporation of three additional factors: the competition by alien species, scenarios on forest management options, and the dispersal abilities of the habitat types’ constituting species. For the latter, supplementary research is necessary to fully comprehend the translation of species’ dispersal into the more complex process of habitat shifts. Moreover, management, such as assisted colonization, enables the establishment of trees even in naturally “unreachable” grid cells.

Expanding the analysis to a continental scale could provide insights into the overall perspectives of the forests, detect large‐scale refugia for the most threatened bog woodlands, and determine Bavaria's role in conserving the selected habitat types. Comparable research is needed to improve the understanding of climate change impacts on other EU habitat types, especially the prioritized ones.

Ultimately, this research addressed essential knowledge gaps regarding the future conservation status of protected EU forest habitat types and the risks they face under climate change. Combining range change analyses based on correlative SDMs with estimates on the coverage by PAs, different levels of exposure of three moisture‐dependent habitats toward climatic changes were identified.

The study therefore offers a methodology for conservation‐oriented research questions in the face of climate change. For the investigated habitat types (*Tilio‐Acerion* forests of slopes, screes, and ravines; bog woodlands; alluvial forests with *A. glutinosa* and *F. excelsior*), first suggestions for conservation strategies were derived. By the second half of the century, practitioners will be confronted with altered climatic conditions of currently established PAs. Environmental suitability maps and exposure comparisons of conservation targets can support them by allocating limited resources to most threatened biota and improving the Bavarian Natura 2000 network under here identified criteria. As the EU law requires favorable conservation statuses for all listed natural habitat types (European Council, 1992), we advocate the evaluation of future impacts on protected habitats to initiate informed conservation strategies.

## CONFLICT OF INTEREST

The authors declare no conflict of interest.

## AUTHOR CONTRIBUTIONS

While all authors conceptualized the study, Claudia Steinacker conducted the analysis, interpreted the results, and led the writing process. Carl Beierkuhnlein and Anja Jaeschke supervised this study and reviewed the manuscript.

## Data Availability

The data used for this study were derived from publicly available datasets. The sources are listed in Appendix S1.2 as well as in the reference list. The R code, produced ensemble models, range change maps, and protected area shapefiles can be accessed under https://doi.org/10.5281/zenodo.3532892 of the Zenodo Repository.
