# Supplementary material for: Assessing the exposure of forest habitat types to projected climate change—Implications for Bavarian protected areas
Source: Ecol Evol. 2019 Nov 28;9(24):14417–29. doi: 10.1002/ece3.5877 (PMC6953681; doi:10.1002/ece3.5877)
Supplement: Supplementary file 1 [file ECE3-9-14417-s001.docx]

**Supplementary Material S1:** Materials for correlative species distribution models.

**Fig. S1.1**: Comparison of observed and modelled current distribution of **(a, b)** *Tilio-Acerion* forests of slopes, screes and ravines, **(c, d)** bog woodlands, and **(e, f)** alluvial forests with *A. glutinosa* and *F. excelsior* in the European Union. Distribution data (resolution: 10x10 km) originate from mandatory monitoring of protected habitat types by member states between 2007-2012. Model projections are based on ensembles of species distribution models, which were built with ‘biomod2’-package for R combining GAM, GLM, GBM, RF.

**Table S1.2**: Utilised data and their corresponding data sources and transformations steps. Final projection was EPSG 3035, ETRS89, LAEA. Resolution for modelling was 1x1 km.

| Dataset | Source | Orig. resol-ution | Pre-processing |
| --- | --- | --- | --- |
| Distribution maps for habitat types | https://www.eea.europa.eu/data-and-maps/data/article-17-database-habitats-directive-92-43-eec-1/distribution-of-species-zipped-shapefile-vector-polygon | 10 km | ▪ extraction of target habitat types  ▪ transformation to grid-cells  ▪ extraction of centroid coordinates |
| Current climate | http://worldclim.org/version2 | 1 km | ▪ reproject  ▪ crop to Europe |
| Future climate | http://www.worldclim.org/cmip5_30s | 1 km | ▪ reproject  ▪ crop to Bavaria |
| Digital elevation model | https://www.eea.europa.eu/data-and-maps/data/eu-dem/dem-epsg-3035/eudem_dem_3035_europe-tif.ovr | 25 m | ▪ resample and mask with climate raster |
| Soil pH at 2m soil depth | http://data.isric.org/geonetwork/srv/eng/catalog.search#/metadata/4c59ee58-a24e-4154-912e-0ff18395ac0d | 250 m | ▪ reproject  ▪ crop to Europe  ▪ resample and mask with climate raster |
| Soil organic carbon content | <http://data.isric.org/geonetwork/srv/ger/catalog.search#/metadata/076db4e8-11a9-4262-b6aa-cfa703a3c0af> | 250 m | ▪ reproject  ▪ crop to Europe  ▪ resample and mask with climate raster |
| Bavarian Natura 2000 areas | <http://www.lfu.bayern.de/gdi/dls/natura2000.xml> | NA | ▪ reproject  ▪ correct invalid geometries |
| Administrative boundaries EU | <https://data.opendatasoft.com/explore/dataset/european-union-countries%40public/export/?sort=admin&location=4,55.47885,21.1377&basemap=mapbox.streets> | NA | ▪ reproject |
| Administrative boundaries Bavaria | <https://opendata.bayern.de/detailansicht/datensatz/verwaltungsgebiete?4> | NA | ▪ reproject |

**Supplementary Material S2:** Procedure of variable selection

Initially, all 19 bioclimatic variables from the WorldClim dataset, as well as five topography related variables (elevation, slope, aspect, “northness”, “eastness”) and four soil parameters (organic carbon content, pH value, soil type and parent material) were integrated into the environmental raster. As the ecology of the three investigated habitat types varies, the following steps were performed for each type separately.

Aiming to find ecologically reasonable, statistically relevant, uncorrelated predictors, a multitude of subsets was created with differing combinations of uncorrelated variables. The subsets experimented with climate-only combinations, expert-knowledge based compositions and purely non-climatic factors. To determine predictors with high explanation power for the observed European distribution of the habitat types, the ‘hier.part’-function in R identified those variables, which reached high importance scores throughout various subsets. Building on this statistical information, the final dataset furthermore considered the known ecological requirements of the key tree species. Therefore, the utilised environmental predictors deviate from the results suggested by the pre-analysis in R.

Due to biomod2-inherent issues with factor-variables, soil type and parent material had to be excluded from the SDMs despite their ecological importance. The topography related variables aspect, “northness” and “eastness” were assigned low explanation power for all three habitat types and consequently were eliminated from the models.

For *Tilio-Acerion* forests, the selected variables were supposed to capture common traits of *Acer pseudoplatanus*, *Tili cordata*, *Tilia platyphyllos, Fraxinus excelsior*, *Ulmus glabra*, *Taxus baccata* and *Corylus avellana*. Among them: the occurrence on slopes and nutrient-rich, moist soils, drought sensitivity, shade tolerance, frost sensitivity and intermediate pH values. Some of these ecological requirements were not included into the environmental raster as reliable spatial datasets are still missing. For example, information on current but especially future soil moisture is not available on continental scale.

The chosen predictors for bog woodlands aimed to represent the required wet, acidic, nutrient poor soil conditions, as well as the cold tolerance of *Picea abies*, *Pinus rotundata*, *Pinus sylvestris*, *Frangula alnus* and *Betula pubescens*. Comparable to missing information on soil moisture, the data availability for nutrients is insufficient for modelling purposes. Expanding the knowledge on environmental conditions throughout Europe is therefore desirable.

Lastly, the variable set for alluvial forests considers the cold sensitivity of *Alnus glutinosa*, as well as the preferred moist to wet soil conditions of further key species (*Fraxinus excelsior*, *Alnus incana*, *Salix alba*, *Populus nigra*, *Betula pubescens*, *Ulmus glabra*). As immediate data on soil moisture was not available, a number of temperature-precipitation relationships were included to indirectly assess how humid a location is.

**Supplementary Material S3:** Statistical results on relationship between local elevational gradients inside of protected areas (PAs) and their modelled future occupation by selected forest habitat types from EU Habitats Directive. For detailed description on models, see methods section and caption of Tab. 2.

**Fig. S3.1**: Elevation range within Natura 2000 areas in Bavaria in relation to their modelled future occupation by habitat types under climate change scenario RCP4.5. Categories “absent” and “present” refer to intersection of modelled distribution of habitat types with Natura 2000 sites. Statistics performed with Wilcoxon Rank Sum and Signed Rank Tests in R package ‘stats’ version 3.5.1.

**Fig. S3.2**: Elevation range within Natura 2000 areas in Bavaria in relation to their modelled future occupation by habitat types under climate change scenario RCP8.5. Categories “absent” and “present” refer to intersection of modelled distribution of habitat types with Natura 2000 sites. Statistics performed with Wilcoxon Rank Sum and Signed Rank Tests in R package ‘stats’ version 3.5.1.

**Fig. S3.3**: Local elevation range within Bavarian Natura 2000 areas in relation to their modelled function as potential hosts for selected forest habitat types under climate change scenario RCP4.5. For detailed description on change classes of PAs and models, see methods section and caption of Tab. 2 and Tab.3. Statistics were performed with Kruskal-Wallis rank sum test and ‘kruskalmc’ post-hoc test (R package ‘stats’ and ‘pgirmess’).

**Supplementary Material S4:** Model projections of environmental suitability for selected forest habitat types in Bavaria, Germany. For detailed description on models, see methods section and caption of Tab. 2. **Figures** (**a, d, g**): weighted mean of occurrence probability according to ensemble model (resolution: 1x1 km); (**b, e, h**): transformed binary projection with threshold that maximises sum of specificity and sensitivity; (**c, f, i**): binary projection aggregated to 10x10 km resolution.

**Figure S4.1**: Modelled current and future distribution of *Tilio-Acerion* forests of slopes, screes and ravines in Bavaria. Threshold for transformation into binary data = 0.52 (ROC cut-off).

**Figure S4.2**: Modelled current and future distribution of bog woodlands in Bavaria. Threshold for transformation into binary data = 0.53 (ROC cut-off).

**Figure S4.3**: Modelled current and future distribution of alluvial forests with *A. glutinosa* and *F. excelsior* in Bavaria. Threshold for transformation into binary data = 0.61 (ROC cut-off).

**Supplementary Material S5:** Evaluation of ensembles of species distribution models.

**Table S5.1**: Performance scores (ROC, TSS, KAPPA) for ensemble models (consensus method: weighted mean) for EU habitat types *Tilio-Acerion* forests of slopes, screes and ravines (9180*), bog woodlands (91D0*) and alluvial forests with *A. glutinosa* and *F. excelsior* (91E0*) modelled on the European scale.

| Performance measure | Habitat type | | |
| --- | --- | --- | --- |
|  | **9180*** | **91D0*** | **91E0*** |
| ROC | 0.930 | 0.968 | 0.930 |
| TSS | 0.696 | 0.794 | 0.696 |
| KAPPA | 0.693 | 0.795 | 0.691 |

**Supplementary Material S6:** Modelled relationship between forest habitat types and the environment. Response curves were built during species distribution modelling with biomod2-pakage utilising model algorithms generalised additive models (GAM), generalised linear models (GLM), generalised boosted methods (GBM) and random forest (RF). Based on reported distribution of habitat types in the European Union.

**Fig. S6.1**: Response curves of ensemble model for *Tilio-Acerion* forests of slopes, screes and ravines to selected environmental variables.

**Fig. S6.2**: Response curves of ensemble model for bog woodlands to selected environmental variables.

**Fig. S6.3**: Response curves of ensemble model for alluvial forests with *A. glutinosa* and *F. excelsior* to selected environmental variables.

**Supplementary Material S7:** Variable importance according to models.

**Table S7.1**: Variable importance of environmental predictors according to ensemble models (consensus method: weighted mean) for EU habitat types *Tilio-Acerion* forests of slopes, screes and ravines (9180*), bog woodlands (91D0*) and alluvial forests with *A. glutinosa* and *F. excelsior* (91E0*) modelled on the European scale. Variable importance was determined with ‘variables_importance‘-function of ‘biomod2‘-package in R.

| Environmental variables | Variable importance for  habitat types | | |
| --- | --- | --- | --- |
|  | ***Tilio-Acerion* forests** | **Bog woodlands** | **Alluvial forests** |
| Minimum temperature of the coldest month | 0.406 | 0.136 |  |
| Temperature annual range | 0.058 | 0.008 | 0.046 |
| Mean temperature of the wettest quarter | 0.060 | 0.035 | 0.175 |
| Mean temperature of the coldest quarter |  |  | 0.352 |
| Precipitation seasonality | 0.055 |  | 0.026 |
| Precipitation of the driest quarter | 0.033 |  |  |
| Precipitation of the warmest quarter |  | 0.015 | 0.172 |
| pH in two meter soil depth | 0.030 | 0.153 | 0.118 |
| Organic carbon content (g/ kg) in two meter soil depth |  | 0.045 | 0.018 |
| Elevation | 0.077 | 0.033 | 0.020 |
| Slope | 0.056 | 0.034 |  |
