# Supplementary figures and images for: Assessing the exposure of forest habitat types to projected climate change—Implications for Bavarian protected areas
Source: Ecol Evol. 2019 Nov 28;9(24):14417–29. doi: 10.1002/ece3.5877 (PMC6953681; doi:10.1002/ece3.5877)

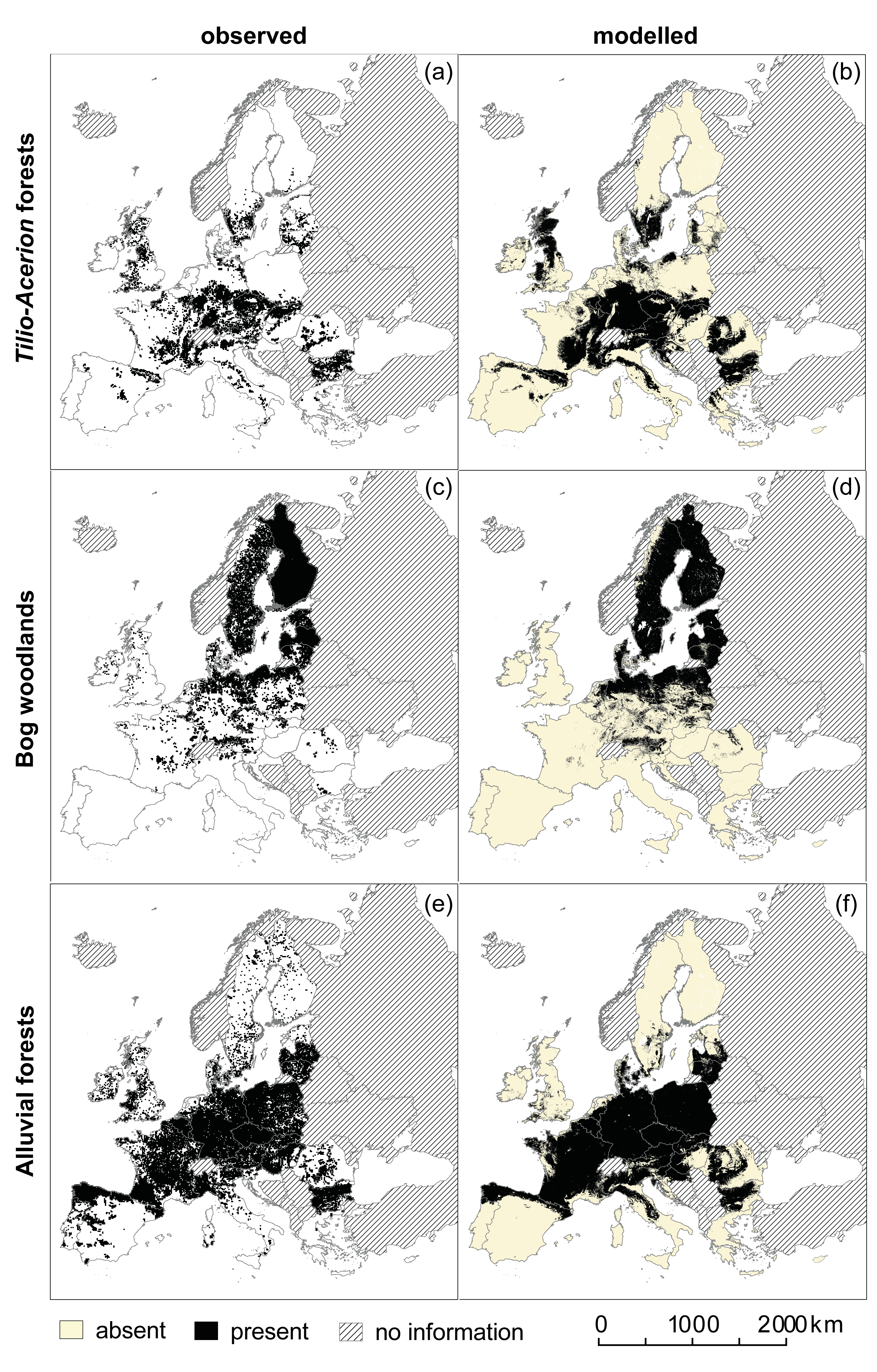

Supplement: Supplementary file 2 [file ECE3-9-14417-s002.tif]

elevation range within PA [m]

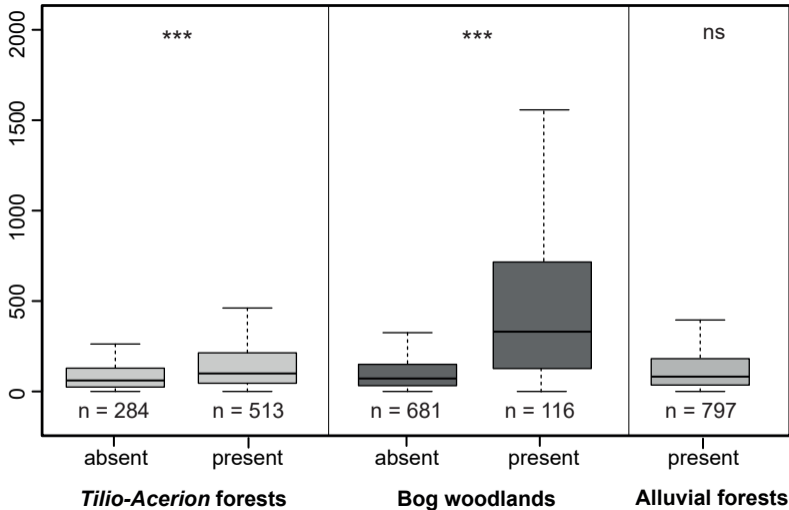

Supplement: Supplementary file 3 [file ECE3-9-14417-s003.pdf]

elevation range within PA [m]

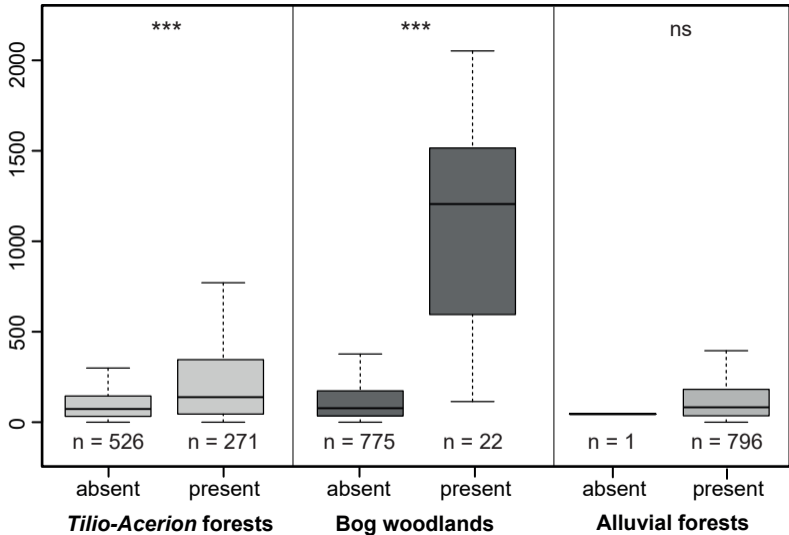

Supplement: Supplementary file 4 [file ECE3-9-14417-s004.pdf]

elevation range within PA [m]

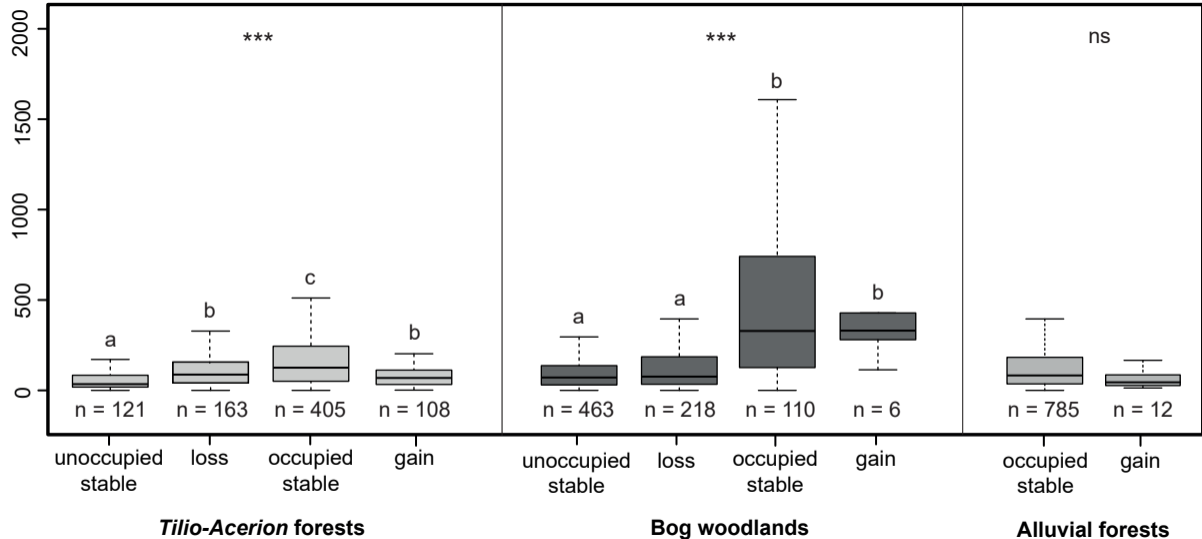

Supplement: Supplementary file 5 [file ECE3-9-14417-s005.pdf]

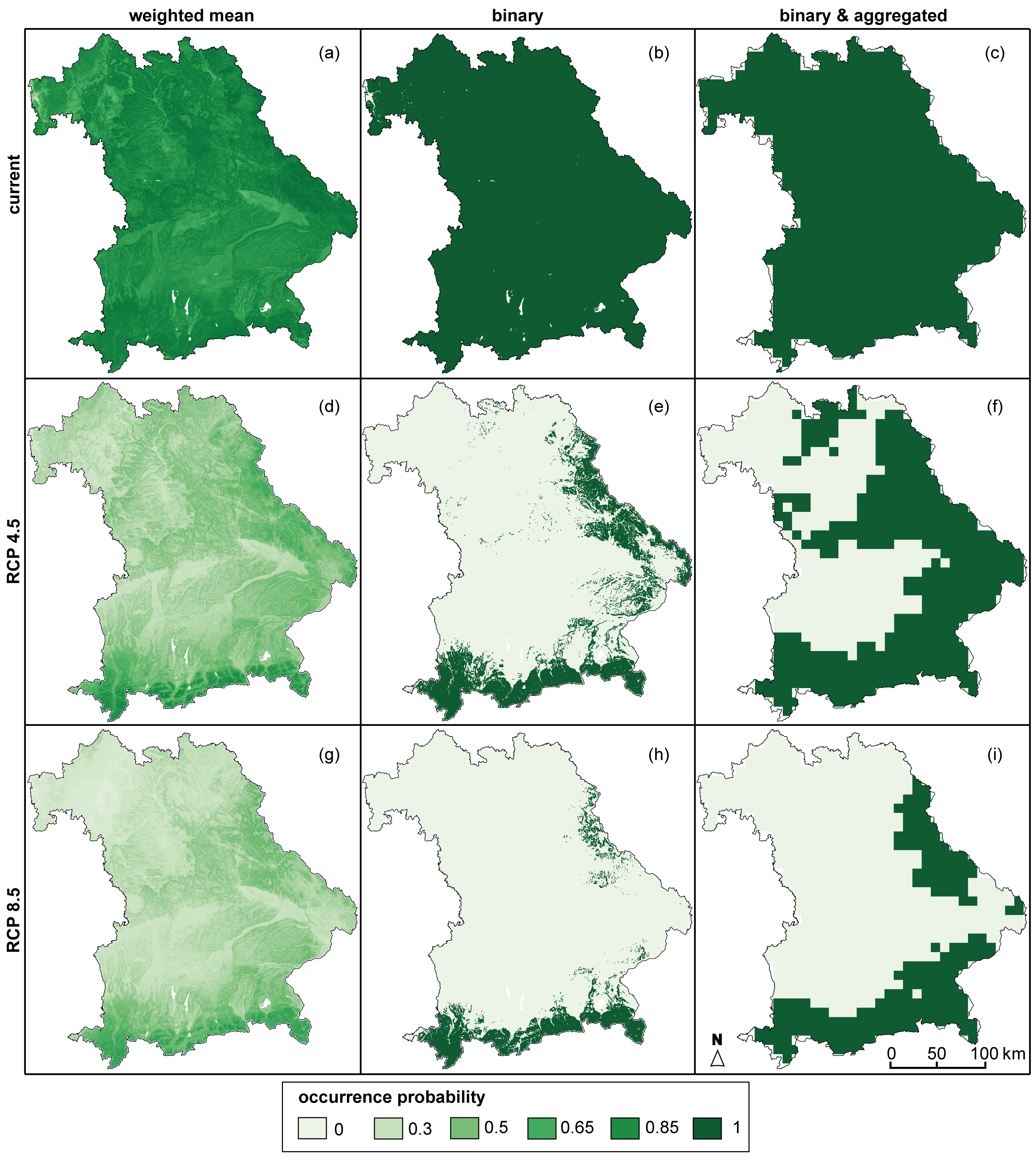

Supplement: Supplementary file 6 [file ECE3-9-14417-s006.tif]

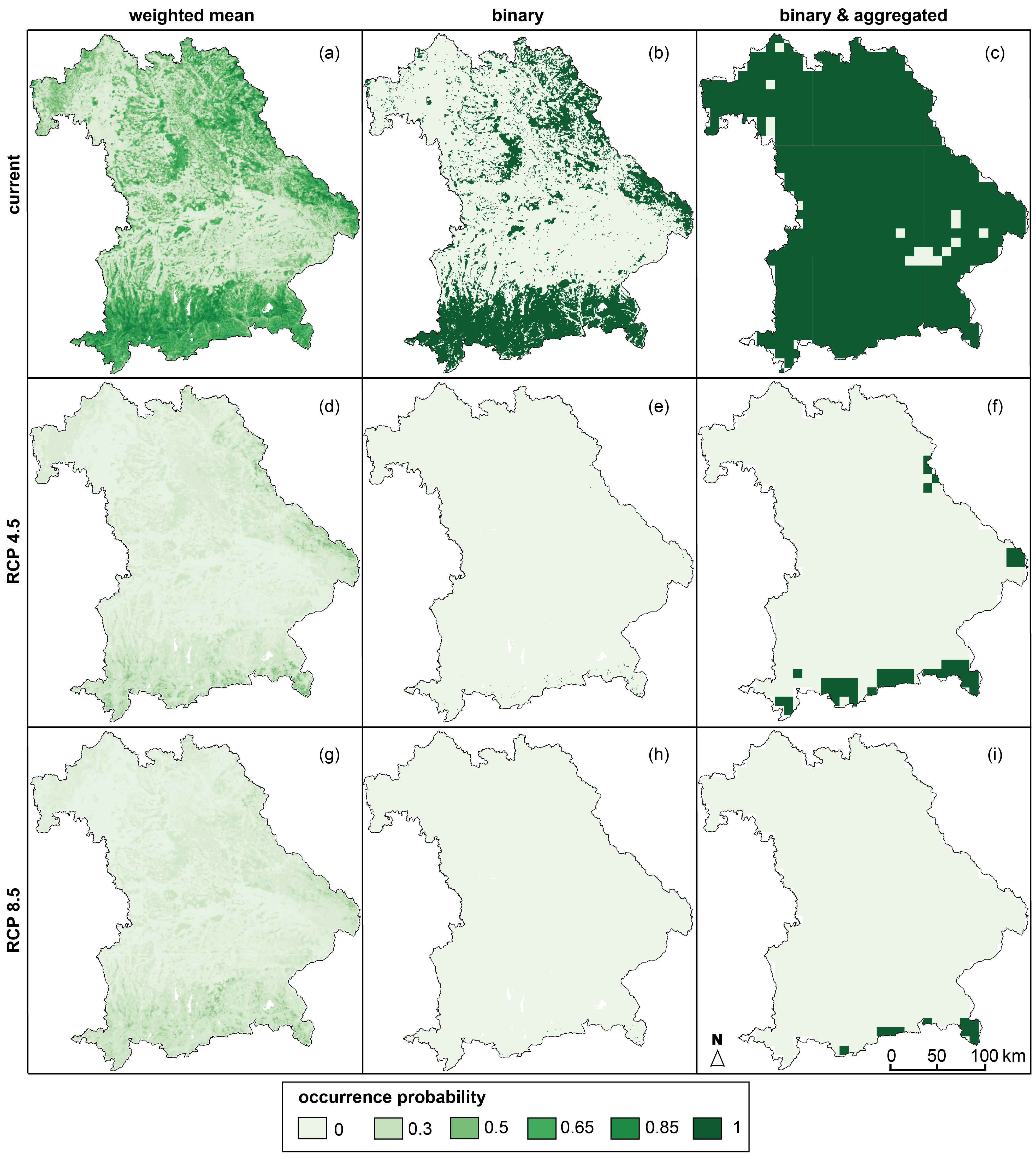

Supplement: Supplementary file 7 [file ECE3-9-14417-s007.tif]

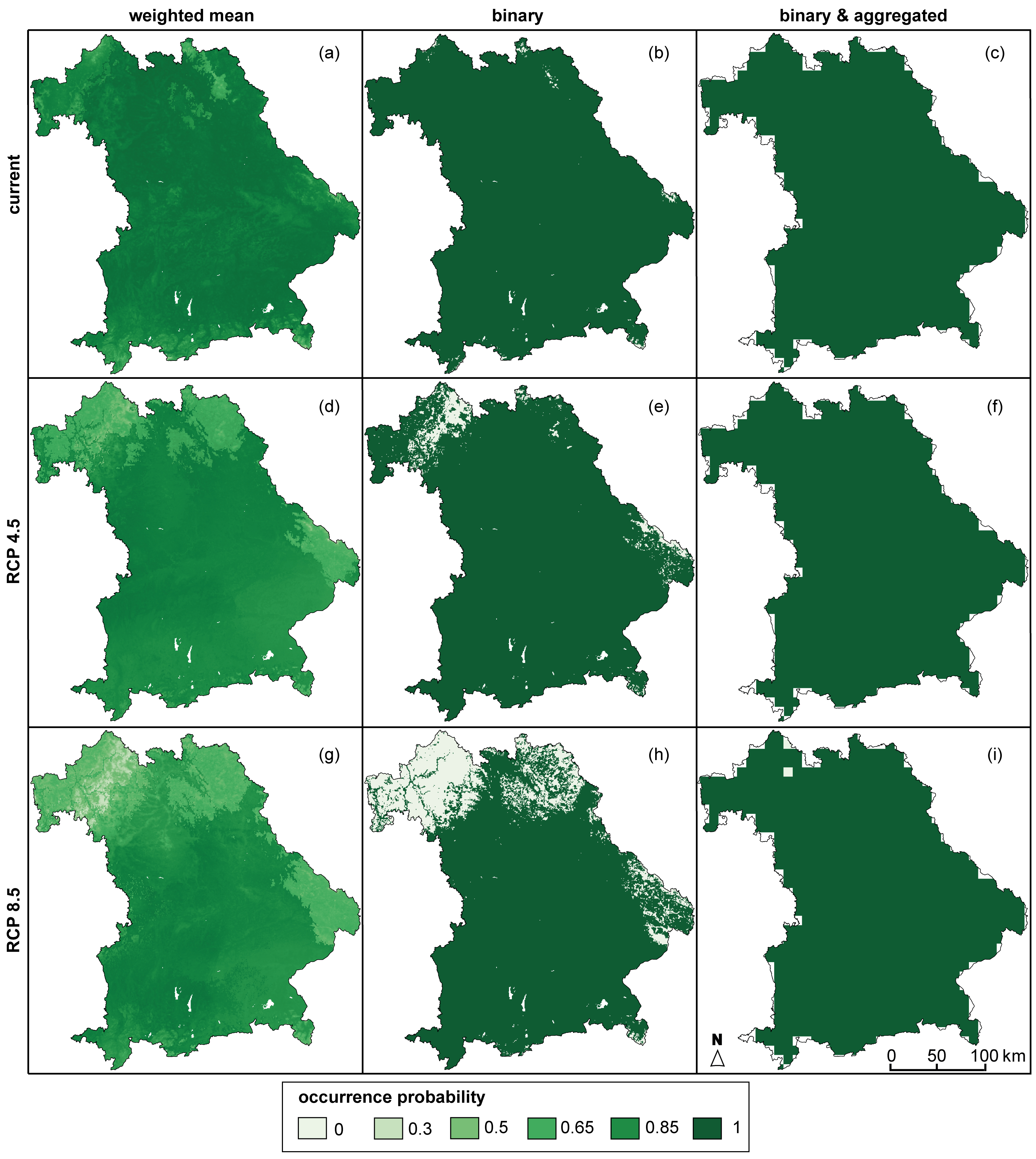

Supplement: Supplementary file 8 [file ECE3-9-14417-s008.tif]
