# Supplementary material for: Assessing the exposure of forest habitat types to projected climate change—Implications for Bavarian protected areas
Source: Ecol Evol. 2019 Nov 28;9(24):14417–29. doi: 10.1002/ece3.5877 (PMC6953681; doi:10.1002/ece3.5877)
Supplement: Supplementary file 9 [file ECE3-9-14417-s009.pdf]

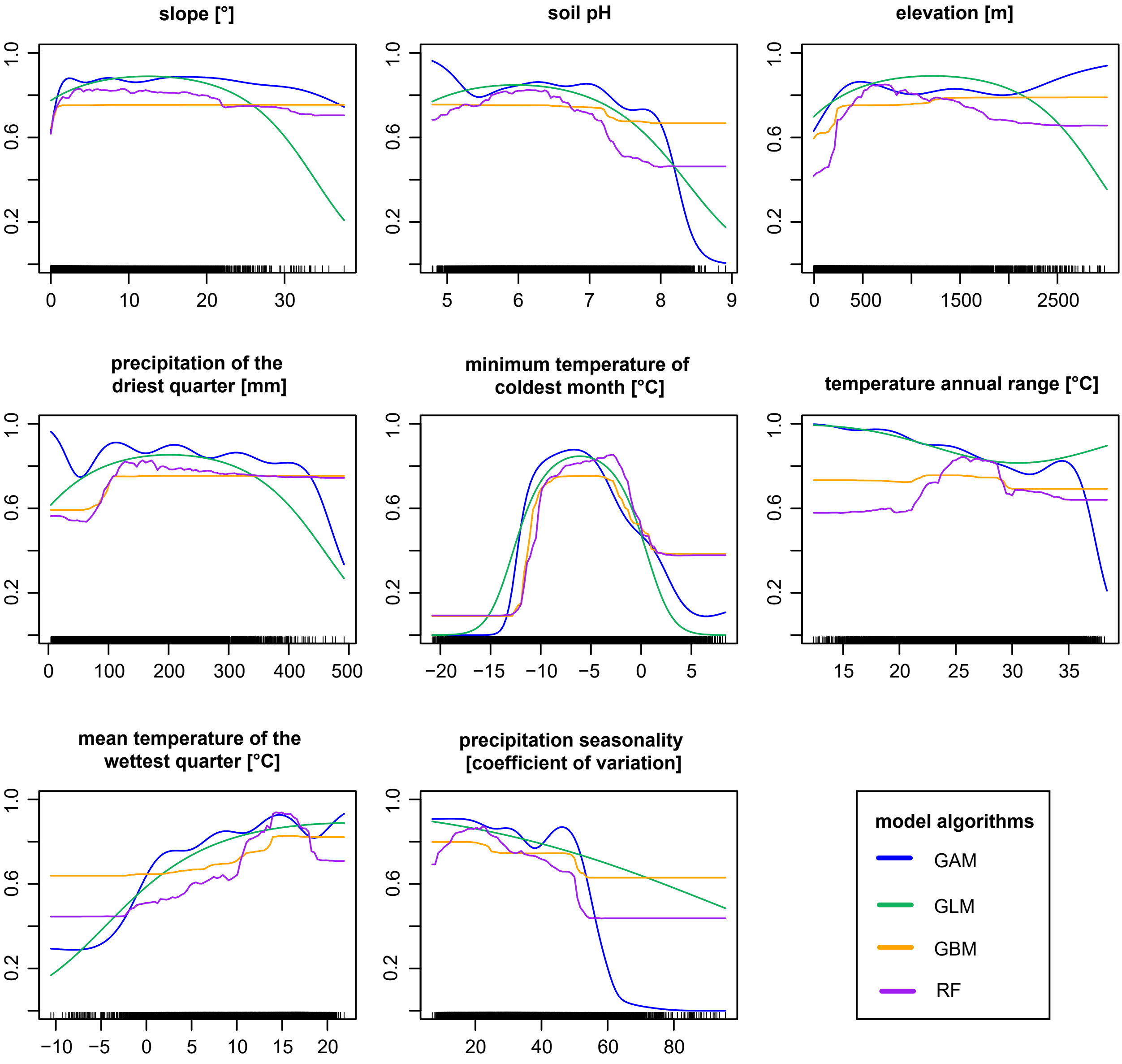
slope [°]
soil pH
elevation [m]
precipitation of the driest quarter [mm]
minimum temperature of coldest month [°C]
temperature annual range [°C]
mean temperature of the wettest quarter [°C]
precipitation seasonality [coefficient of variation]
0.2
0.6
1.0
0
10
20
30
5
6
7
8
9
500
1500
2500
100
200
300
400
500
−20
−15
−10
−5
5
15
25
35
40
60
80
model algorithms
GAM
GLM
GBM
RF
