# Supplementary material for: Assessing the exposure of forest habitat types to projected climate change—Implications for Bavarian protected areas
Source: Ecol Evol. 2019 Nov 28;9(24):14417–29. doi: 10.1002/ece3.5877 (PMC6953681; doi:10.1002/ece3.5877)
Supplement: Supplementary file 10 [file ECE3-9-14417-s010.pdf]

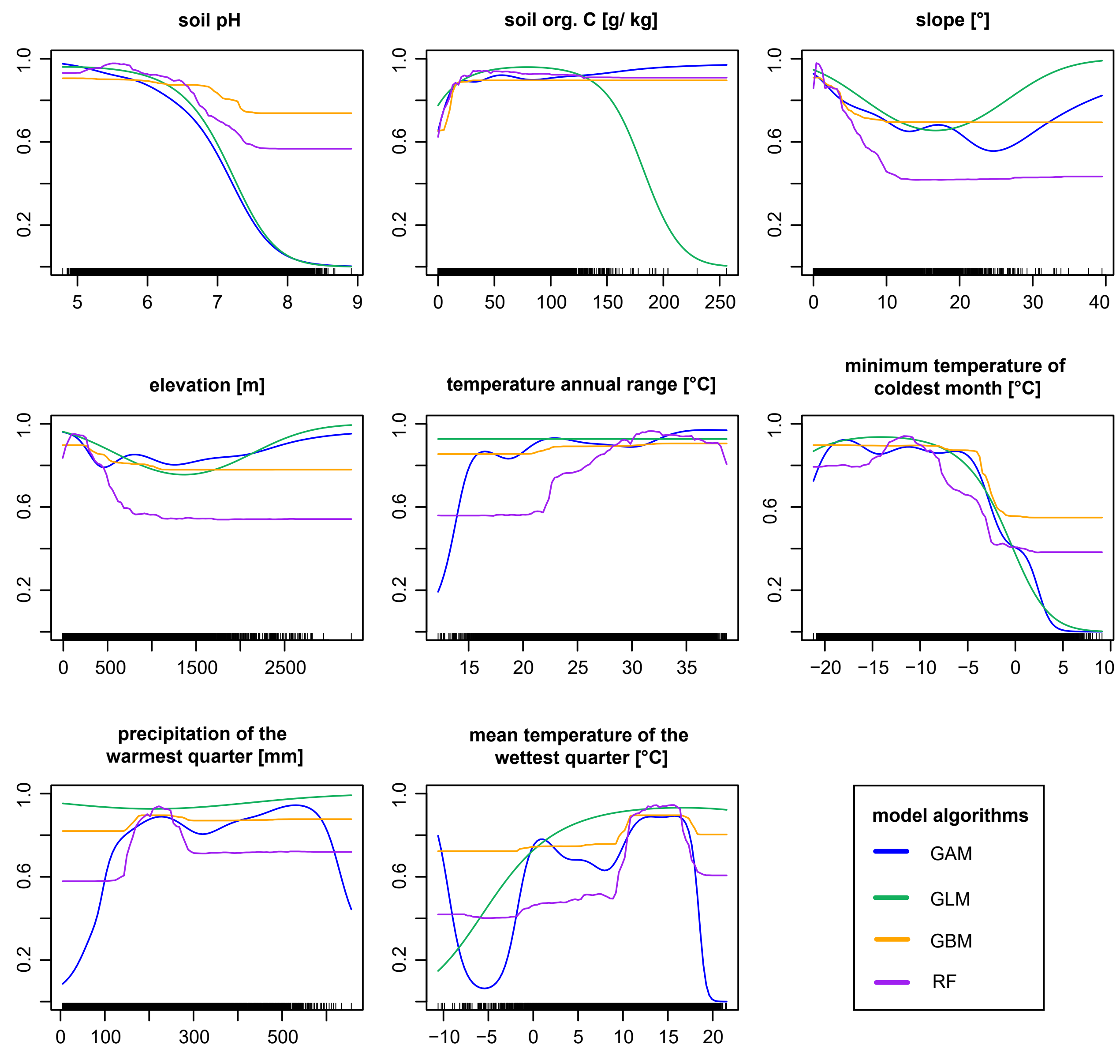

soil pH
soil org. C [g/ kg]
slope [°]
elevation [m]
temperature annual range [°C]
minimum temperature of coldest month [°C]
precipitation of the warmest quarter [mm]
mean temperature of the wettest quarter [°C]
model algorithms
GAM
GLM
GBM
RF
