# Supplementary material for: Assessing the exposure of forest habitat types to projected climate change—Implications for Bavarian protected areas
Source: Ecol Evol. 2019 Nov 28;9(24):14417–29. doi: 10.1002/ece3.5877 (PMC6953681; doi:10.1002/ece3.5877)
Supplement: Supplementary file 12 [file ECE3-9-14417-s012.pdf]

Dataset	Source	Orig. resol-ution	Pre-processing
**Distribution maps for habitat types**	https://www.eea.europa.eu/data-and-maps/data/article-17-database-habitats-directive-92-43-eec-1/distribution-of-species-zipped-shapefile-vector-polygon	10 km	▪ extraction of target habitat types<br>▪ transformation to grid-cells<br>▪ extraction of centroid coordinates
**Current climate**	http://worldclim.org/version2	1 km	▪ reproject<br>▪ crop to Europe
**Future climate**	http://www.worldclim.org/cmip5_30s	1 km	▪ reproject<br>▪ crop to Bavaria
**Digital elevation model**	https://www.eea.europa.eu/data-and-maps/data/eu-dem/dem-epsg-3035/eudem_dem_3035_europe-tif.ovr	25 m	▪ resample and mask with climate raster
**Soil pH at 2m soil depth**	http://data.isric.org/geonetwork/srv/eng/catalog.search#/metadata/4c59ee58-a24e-4154-912e-0ff18395ac0d	250 m	▪ reproject<br>▪ crop to Europe<br>▪ resample and mask with climate raster
**Soil organic carbon content**	http://data.isric.org/geonetwork/srv/ger/catalog.search#/metadata/076db4e8-11a9-4262-b6aa-cfa703a3c0af	250 m	▪ reproject<br>▪ crop to Europe<br>▪ resample and mask with climate raster
**Bavarian Natura 2000 areas**	http://www.lfu.bayern.de/gdi/dls/natura2000.xml	NA	▪ reproject<br>▪ correct invalid geometries
**Administrative boundaries EU**	https://data.opendatasoft.com/explore/dataset/european-union-countries%40public/export/?sort=admin&location=4,55.47885,21.1377&basemap=mapbox.streets	NA	▪ reproject
**Administrative boundaries Bavaria**	https://opendata.bayern.de/detailansicht/datensatz/verwaltungsgebiete?4	NA	▪ reproject
