# Supplementary material for: Assessing the exposure of forest habitat types to projected climate change—Implications for Bavarian protected areas
Source: Ecol Evol. 2019 Nov 28;9(24):14417–29. doi: 10.1002/ece3.5877 (PMC6953681; doi:10.1002/ece3.5877)
Supplement: Supplementary file 13 [file ECE3-9-14417-s013.pdf]

Performance measure	Habitat type		
	9180*	91D0*	91E0*
ROC	0.930	0.968	0.930
TSS	0.696	0.794	0.696
KAPPA	0.693	0.795	0.691
