# Supplementary material for: Assessing the exposure of forest habitat types to projected climate change—Implications for Bavarian protected areas
Source: Ecol Evol. 2019 Nov 28;9(24):14417–29. doi: 10.1002/ece3.5877 (PMC6953681; doi:10.1002/ece3.5877)
Supplement: Supplementary file 14 [file ECE3-9-14417-s014.pdf]

Environmental variables	Variable importance for habitat types		
	Tilio-Acerion forests	Bog woodlands	Alluvial forests
Minimum temperature of the coldest month	0.406	0.136	
Temperature annual range	0.058	0.008	0.046
Mean temperature of the wettest quarter	0.060	0.035	0.175
Mean temperature of the coldest quarter			0.352
Precipitation seasonality	0.055		0.026
Precipitation of the driest quarter	0.033		
Precipitation of the warmest quarter		0.015	0.172
pH in two meter soil depth	0.030	0.153	0.118
Organic carbon content (g/ kg) in two meter soil depth		0.045	0.018
Elevation	0.077	0.033	0.020
Slope	0.056	0.034	
